# Fruit and Vegetable Consumption Among Adolescent Secondary School Students in Boukombe and Natitingou, North Benin

**DOI:** 10.3390/ijerph22050767

**Published:** 2025-05-13

**Authors:** Melina Maureen Houndolo, Sam Bodjrenou, Irmgard Jordan, Elianna Majaliwa, Elie Koukou, Kandala Ngianga-Bakwin, Colette Azandjeme, Melanie Nyambura Katsivo, Céline Termote, Waliou Amoussa Hounkpatin

**Affiliations:** 1 International Center for Tropical Agriculture (CIAT), Cotonou 08 P.O. Box 932, Benin; s.bodjrenou@cgiar.org (S.B.); eliekoukou@gmail.com (E.K.); 2 Department of Nutrition and Food Sciences Abomey-Calavi, Faculty of Agricultural Sciences, University of Abomey-Calavi, Abomey Calavi 01 P.O. Box 526, Benin; majaliwaalisa@gmail.com (E.M.); waliou.amoussah@uac.bj (W.A.H.); 3 Department of Epidemiology and Biostatitics, Schulich School of Medicine & Dentistry, University of Western Ontario, London, ON N6A 5C1, Canada; nkandala@uwo.ca (K.N.-B.); mkatsivo@uwo.ca (M.N.K.); 4 Bioversity International, Nairobi P.O. Box 823-00621, Kenya; i.jordan@cgiar.org (I.J.); c.termote@cgiar.org (C.T.); 5 Institut Régional de Santé Publique, Ouidah P.O. Box 384, Benin; colsyaz@yahoo.fr

**Keywords:** adolescents, fruit and vegetables, secondary school students, Boukombe, Natitingou, micronutrient deficiencies

## Abstract

Fruit and vegetables (F&V) are recommended for a healthy life. Adolescence is a critical period for the onset of eating disorders and future health. F&V consumption among adolescents is globally low, making this group a key target for diet/nutrition-related interventions. This cross-sectional study aimed to assess F&V consumption among secondary school students in the food-insecure communes of Boukombe (rural) and Natitingou (urban), Benin. Using probabilistic random sampling, 303 students completed F&V intake frequency questionnaires and 24-h dietary recalls on school and non-school days. Poisson models identified factors associated with F&V consumption. The results showed that only 8.8% (Boukombe) and 11% (Natitingou) of students consumed fruit at least twice per day, and over 80% of students had not eaten fruit in the preceding 24 h; 9.9% and 11.4%, respectively, consumed vegetables at least twice per day. On average, 45.5% of students in Boukombe and 68% in Natitingou consumed at least three types of vegetables on school days. The most commonly consumed fruits were oranges in Boukombe and lemons in Natitingou. Factors influencing fruit consumption included sex (*p* = 0.005), age (*p* = 0.04), and mothers’ occupation (*p* = 0.03) on school days/and school or non-school days, while commune (*p* = 0.00017) and ethnic group affected vegetable consumption. Such low F&V consumption among surveyed students is a matter of public health concern, as it is likely to affect their health―in terms of micronutrient deficiency―and intellectual performance. These results should incentivize nutrition researchers, project managers, public health officials, and policymakers to (re)design and implement broader measures targeting secondary school students’ dietary practices to increase their F&V consumption.

## 1. Introduction

Ensuring good nutrition and achieving food security are the major challenges of our generation [1]. Indeed, malnutrition and food insecurity are still public health problems in most low- and middle-income countries [2,3]. In Benin, as in many other African countries, food insecurity remains a serious problem, with more than one in four (26%) households being food insecure [4,5]. Atacora, in northern Benin, is one of the departments most affected by food insecurity and malnutrition, with 24% of the population being food insecure [6]. The food and nutrition assessment indicator of stunting prevalence among under-five-year-old children was 33% in 1996 and remained stable at 32% until 2018 [7]. This is above the critical Public Health level of 30% defined by the World Health Organization [8]. The prevalence of food insecurity within Benin households varies from one commune to another within the department, where the highest values are observed in the communes of Boukombe, Toucoutouna, and Natitingou with 46.3%, 30%, and 26.8%, respectively [4].

The department of Atacora has a predominantly adolescent population aged between 10 and 19 years [9], and school is one of the places adolescents spend most of their day. Adolescence is a critical period, i.e., often the time of life for the onset of eating disorders [10]. Adolescents are thus an important target group for intervention efforts aiming to improve dietary practices [11,12,13]. Across the world, adolescents have or develop poor eating habits and unhealthy diets [14,15]. The best way to avoid an unhealthy diet, with long-term effects, is to promote a diverse, healthy diet through the consumption of fruit and vegetables (F&V), which are generally considered two important components of a healthy diet [16].

Fruit and vegetable consumption significantly influences health and reduces the risk of disease incidence, including non-communicable diseases [17]. A diet rich in F&V is recommended for human well-being at any age, and in particular for adolescents [18], as they provide the body with vitamins, minerals, fiber, and phytochemicals necessary for growth and development, cognitive performance, and health maintenance [19]. The World Health Organization (WHO) recommends a daily F&V intake of 400 g, or five daily portions of F&V [20], yet a large proportion of adolescents and secondary school students worldwide do not meet these recommendations [17]. Note: This study focuses on adolescents in secondary school education in Benin, aged 11–19. In the narrative, for simplicity, we refer to the study cohort as ‘students’ or secondary-school students’. Table 1 summarizes the results of some relevant studies assessing the levels of F&V consumption among secondary school students in different countries.

There is scant information about F&V consumption among students/adolescents in Benin [24,25] and, to our knowledge, no study has been conducted in the department of Atacora, which is the most affected by food insecurity. However, there is a need for accurate data on young people (vulnerable groups) from this region to explore the determining factors associated with poor F&V consumption, to identify and tailor strategies to improve F&V consumption among these adolescents. This study thus aimed at assessing F&V consumption among adolescent secondary school students in two schools of Boukombe and Natitingou and the factors associated with these consumption rates. The results of this study will supply important information on this age group to assess the need for school-based nutrition interventions in Benin’s secondary schools.

## 2. Materials and Methods

### 2.1. Study Area

This study was conducted in the department of Atacora in Northern Benin, specifically in the communes of Boukombe and Natitingou. Atacora department has a population of 769,337 residing within nine communes. Natitingou commune is the main city in the department. Boukombe and Natitingou communes are characterized by agricultural, forestry, and pastoral livelihoods [26]. Boukombe is a rural commune, while Natitingou is urban. Both Boukombe and Natitingou have 46.3% and 26.3% of food-insecure households, respectively. Agriculture is the main economic activity, often involving slash-and-burn, with other traditional techniques predominating. The most widely cultivated crops are maize, millet, cassava, and fonio [27]. Livestock breeding is the second main activity, mainly characterized by cattle, sheep, goats, pigs, poultry, and dogs [27].

### 2.2. Sampling and Participant Recruitment

In this cross-sectional, descriptive, and analytical study, we used convenience and probability random sampling. Convenience sampling was used to select the secondary schools, while probability random sampling was used to select the students. The target population was secondary or high school students aged between 11 to 19, enrolled in schools in the two selected communes. Overall, four and 11 public secondary schools were identified in Boukombe and Natitingou communes, respectively. In each commune, the largest public secondary school was selected, based on the number of students enrolled.

**Figure 1:**
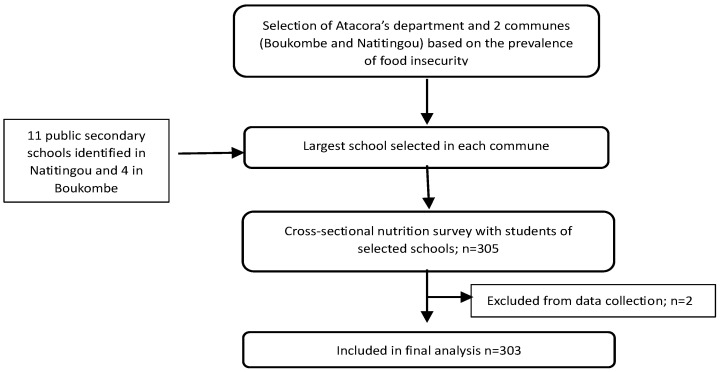
Study flow, including selection and dropouts.

The sample size was calculated using the following formula [28]:

N = Z^2^ × p (1 − p)/m^2^

with Z being the confidence level (typical value of 1.96); p the prevalence of low dietary diversity in Atacora (which is 22.4% [6]), and m a 5% margin of error (standard value of 0.05). The value for dietary diversity was taken from household surveys, as there was no specific data related to adolescents’ dietary diversity in this area. The sample size calculation resulted in a sample size of 268 students. Considering a 10% rate of refusal and non-response, the final sample size consisted of 303 students in total, randomly selected from the two secondary schools. A proportional distribution was made according to the number of enrolled students in each school to ensure a representative sample. The selection criteria were for students to: (i) be enrolled in a non-exam class of the selected secondary schools; (ii) have received their parents’ approval to participate in the study; (iii) willingly participate in the study; and (iv) be available for interview both on weekdays and weekends. After receiving the approval of the schools’ directors, the students were first met by the study management team in their respective classes. At this first meeting, the study’s goal was explained, followed by a questions and answers session. After that, 318 students volunteered to participate in the study, from which 305 students were randomly selected. The selected students met for a second time to complete the participant consent form. Out of the 305 selected students, two students were not available and were consequently excluded. In the end, 303 students participated (101 students in Boukombe’s secondary school, where 1150 students were enrolled, and 202 students in Natitingou’s secondary school, where 2350 students were enrolled at the time of data collection) and individual interviews were conducted by administering three different questionnaires (Figure 1).

### 2.3. Enumerator Training

Data collection was carried out from 18 November to 21 December 2023. Before starting, enumerators were selected based on their data collection experience and proficiency in the study areas’ local languages. Enumerators were trained in the different tools and a pre-test was conducted to assess the tools’ and the enumerators’ efficiency. Data were collected using Kobo Collect and Kobo toolbox software [29,30,31].

### 2.4. Data Collection

#### 2.4.1. Socioeconomic Data

To characterize the students, their socio-economic data were collected [32]. The students’ age was calculated from their birthdate and recorded in years. Students’ sex, ethnic origin, and fathers’ and mothers’ principal sources of income were also recorded.

#### 2.4.2. Fruit and Vegetable Consumption Frequency Data

Data relating to F&V consumption frequency were collected to assess usual F&V consumption based on different modalities―once a day, two or more times a day, 3–4 times a week, 5–6 times a week, 1–3 times a month, less than once a month or never)―as suggested by [33].

#### 2.4.3. 24-h Dietary Recall Data

Finally, an interview based on a multiple-pass qualitative 24-h dietary recall questionnaire was conducted to reduce bias due to memory [34,35,36]. This method consists of five steps: (1) the ‘quick list’, which is an uninterrupted listing by the participant of foods and beverages consumed in the 24 h preceding the interview; (2) the forgotten-foods list, which queries the participant on categories of foods that have been documented as frequently forgotten; (3) listing the times and occasions when foods were consumed; (4) the detail cycle, which elicits descriptions of foods and amounts eaten; and (5) the final probe review [37,38]. For this questionnaire, two rounds of 24-h dietary recall were conducted on non-consecutive days―a school day and a non-school (weekend) day [39]. This was done to obtain an overview of the students’ F&V consumption, even though it would not provide us with the students’ actual micronutrient intakes.

### 2.5. Data Cleaning and Processing

After collection, the data were extracted from Kobo to an Excel tab for cleaning and processing. Duplicate entries were deleted. Overall, 12 duplicate entries in the 24-h recall base and data from six participants who did not complete two 24-h recalls were deleted before processing.

The parents’ occupations were categorized into nine groups: Farmer, Breeder, Public service worker, Taxi driver, Trader, Craftsman, Private service worker, Caregiver, Processor, and No occupation.

In order to assess the adequacy of students’ F&V consumption, data from the F&V consumption frequency questionnaire responses were used. At this point, students who consumed F&V at least twice a day were considered to have an adequate F&V intake, and all the remaining respondents were considered to have an inadequate intake [23]. From the 24-h dietary recall data, we extracted an exhaustive list of different F&V consumed by students on school and non-school days. To determine the percentage of students who consumed each fruit or vegetable, a score of ‘1’ was given when the fruit or vegetable was consumed by the student and ‘0’ if not. F&V scores were calculated to assess the most-consumed F&V, as well as to determine the total number of F&V consumed per student in the previous 24 h. With the two sets of 24-h dietary recall data, we also appraised whether the number of different F&V consumed by students on school days differed from what they consumed on non-school days.

### 2.6. Data Analysis

Data were analyzed using R software version 4.1.3. Descriptive analyses, including frequency and proportion, were conducted for all variables. The chi-square test and Fisher’s test were used to assess the distribution and association between the categorical variables. The Shapiro test was used to check the normality of the distribution of quantitative variables before applying the appropriate comparison tests between the means of the quantitative variables. Levene’s test was used to assess the homogeneity of variances between the Natitingou and Boukoumbe student populations for fruit consumption frequency; vegetable consumption frequency; fruits consumed; vegetables consumed; and students’ sex, age, ethnic group, and fathers’ and mothers’ occupation. Where the data did not follow a normal distribution, Wilcoxon–Mann–Whitney’s non-parametric test was used to compare the distributions of the two groups of individuals, based on the medians of the quantitative variables. A Poisson model with zero inflation was used to determine the factors influencing the number of F&V consumed. This is because the dependent variable followed a Poisson distribution with zero inflation, indicating count data with a predominance of zeros (in the case of fruit consumption). A negative binomial model was used as an alternative to the Poisson model. In addition, the Poisson generalized estimating equations (GEE) model was used to account for the correlation between observations in the count data. A significance level of 5% (*p* < 0.05) was considered.

## 3. Results

### 3.1. Participants’ Socio-Economic Data

The participating students’ socio-economic background data are presented in Table 2. Students’ average age was 15.88 ± 2.72 years in Boukombe and 15.32 ± 2.72 years in Boukombe. In this study, more girls than boys participated in both sites (59.39% in Natitingou and 56.57% in Boukombe). In terms of the main source of parental income, 45% of students’ fathers in Boukombe reported agriculture as their main occupation, while in Natitingou, 38% were public service workers. Mothers in Boukombe were more frequently categorized under “caregiver” (45.45%), while those in Natitingou were more commonly traders (41.11%) (Table 2).

### 3.2. Fruit and Vegetable Consumption Frequency Based on the F&V Frequency Consumption Questionnaire

In the Boukombe secondary school, only 8.8% of students had reported consuming fresh fruits at least twice a day, as did 11% of Natitingou’s students (*p* = 0.01). Only 9.9% of Boubombe’s students and 11.44% of Natitingou’s students had reported consuming vegetables at least twice a day (Table 3).

### 3.3. Number of Fruits and Vegetables Consumed Based on 24-h Dietary Recall

Fruits: The percentage of students consuming at least two different fruits in the Boukombe secondary school was 2.02% on school days and 4.04% on non-school days (*p* = 0.33), which was lower compared to the Natitingou secondary school, where 5.06% of students consumed at least two different fruits on school days and 5.14% on non-school days (*p* = 0.06). The difference between the two sites is significantly different (*p* = 0.034) (Table 4).

Vegetables: There was a lower prevalence of students consuming at least three different vegetables per day—45.45% and 55.55% on school and non-school days, respectively—in Boukombe, compared to the Natitingou secondary school, where it was 67.99% and 66.46% on school and non-school days, respectively. There was no significant difference between the different types of days (Boukombe *p* = 0.24; Natitingou *p* = 0.65), but a significant difference between the two secondary schools was observed (*p* < 001).

### 3.4. Fruits Consumed Based on 24-h Recall

**Figure 2:**
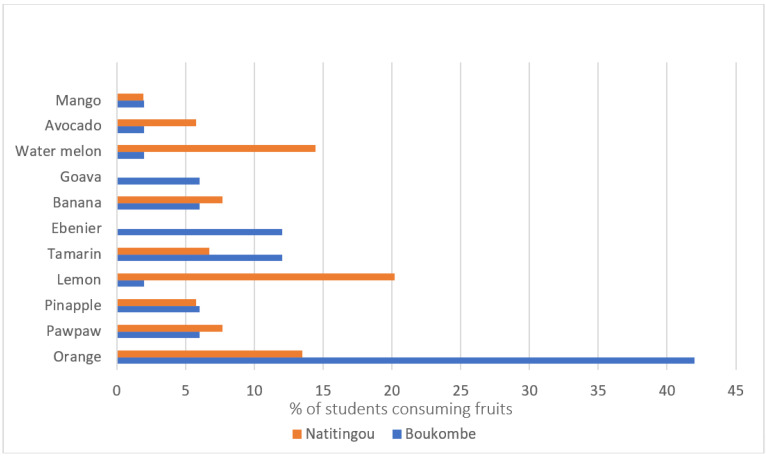
The diversity of fruits consumed in Boukombe and Natitingou by secondary school students.

Eleven different fruits were consumed by the students in both secondary schools. The most-consumed fruit in the Boukombe secondary school was oranges (42%), while in the Natitingou secondary school it was lemons (consumed as juice) (20.19%) (Figure 2). Hibiscus-flower juice/tea was also identified as a beverage consumed by students but was not counted among the 11 fruits reported as consumed in the 24-h recall.

### 3.5. Vegetables Consumed Based on 24-h Recall

**Figure 3:**
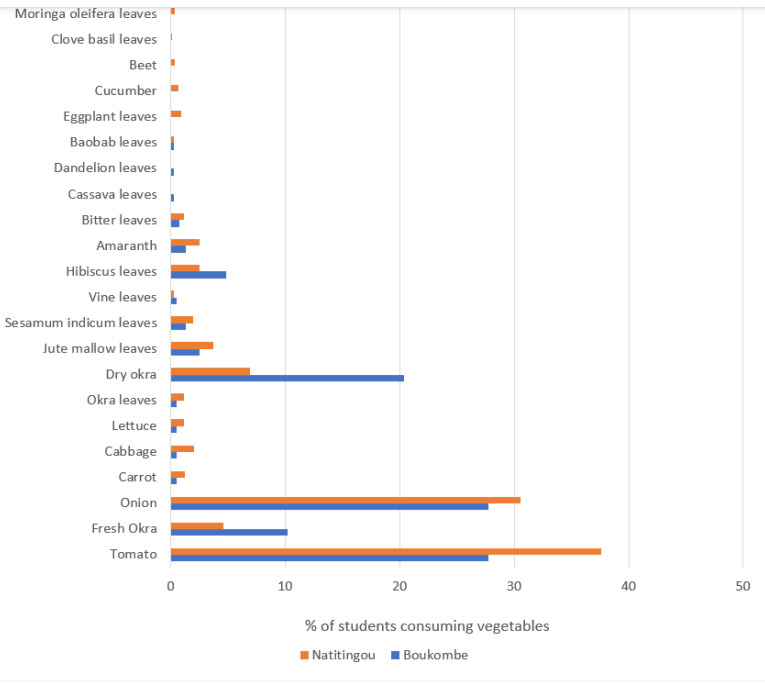
Vegetables consumed in Boukombe and Natitingou by secondary school students.

The 24-h recall identified 22 different vegetables being consumed by Natitingou and Boukombe secondary school students (Figure 3)—the most important being tomatoes (Boukombe 27.73% and Natitingou 37.58%) and onion (Boukombe 27.73% and Natitingou 30.57%), followed by dry okra (Boukombe 20.35% and Natitingou 6.89%).

### 3.6. Factors Associated with Fruit Consumption

Students’ sex (*p* = 0.005), age (years) (*p* = 0.04), and mothers’ occupation (*p* = 0.03) significantly influenced students’ fruit consumption on school days. On non-school days, students’ ethnicity (*p* = 0.001) and mothers’ occupation (*p* = 0.039) significantly influenced students’ fruit consumption (Table 5).

### 3.7. Factors Associated with Vegetable Consumption

Factors identified as being associated with vegetable consumption included students’ commune (*p* = 0.00017) and their ethnicity (Table 6).

## 4. Discussion

This study has revealed a very low consumption of F&V among students from both targeted secondary schools in Boukombe and Natitingou communes. Most of the students did not meet the WHO daily intake recommendation of five varieties of fruits and vegetables per day or a total of 400 g of F&V daily [20]. This implies that, in these schools, more than three out of four (>75%) students may suffer from micronutrient deficiencies. Regular fruit consumption is recognized as having a tangible effect on well-being, as well as on brain function [40], implying that low fruit consumption can affect students’ health and their school performance. A cross-sectional study using a food frequency questionnaire carried out in Burkina Faso on secondary school students showed that 75.4% of students ate less than one serving of fruit per day [41]. Another study, based on the Laos Global School-Based Student Health Survey (GSHS), found a prevalence of 74.0% for inadequate fruit consumption [42], which is 20% lower than in our study. The mean daily consumption of fruit identified in a study for Bangladeshi secondary school students was 1.22 servings [22]. These studies have shown similar F&V consumption rates, indicating a severe challenge in F&V consumption levels for this age group in many food-insecure countries. The low prevalence of fruits consumed in both Boukombe and Natitingou may be due to their location within the most insecure food department of Benin [4] and also limited seasonal fruit availability at the time of year when the data were collected, as fruit availability can fluctuate based on the season [43]. The most consumed fruit, oranges in Boukombe’s secondary school, and lemons (consumed as juice) in Natitingou’s secondary school, could also be associated with the data collection period. The orange season in Benin is between September and December, which is when our data were collected [44].

Students consumed more vegetables than fruits. This aligns with a study carried out in the Arab States, which also found that fruits were less consumed than vegetables [21], with mean daily consumption of fruits being 1.22 servings, while for vegetables it was 1.99 servings [22]. Our study indicates that the most consumed vegetables in both secondary schools were tomatoes and onions. This may be because tomatoes and onions are among the most common vegetables used in most daily meals, and they can easily be obtained throughout the year. Dry okra—the third most consumed vegetable in both secondary schools—is often produced during harvesting periods to reduce waste and loss. It is used as a base for sauces in the dry season when fresh okra is not available or too expensive. This is an adaptation strategy for people who cannot afford fresh okra, which allows them access to vegetables albeit dried. Students’ diets at our study sites lacked African green leafy vegetables, which are a very important source of micronutrients for health and well-being [45]. Similar results have been found with primary school children in Nigeria and also among secondary school students in Australia [46,47].

Our study revealed that students’ sex, age, and their mother’s occupation influenced fruit consumption, while ethnic group and commune was shown to influence vegetable consumption. We also found that boys ate more fruit than girls. This could be explained by the fact that, in both our study areas, many fruit trees are publicly accessible and boys may be more able or inclined to climb fruit trees than girls. Conversely, a cross-sectional study in Iran among adolescents showed that girls consumed more fruits than boys [18]. In our study areas, students whose mothers earned an income also consumed more fruit than other students. Similar results are shared by other studies, showing that fruit consumption is higher among students with income-generating mothers and/or with higher educational levels [48,49,50]. Mothers are often responsible for family feeding practices and the wealthier they are, the more they can improve their children’s dietary practices and make better food choices for their family. A significant difference was found between vegetable consumption in Boukombe (rural area) and Natitingou (urban area), where the Boukombe rural students consumed fewer vegetables than the Natitingou urban students. Conversely, a study conducted in Brazil found that students living in rural areas ate more vegetables than those living in urban areas [51]. This difference could be explained by our data having been collected in the ‘transition period’, a time of the year when Boukombe normally faces a water shortage, limiting F&V production. Farmers in these communes only have access to sufficient water during the rainy season. As Natitingou is an urban area, it does not face these issues, and the farmers of nearby villages bring their produce to Natitingou to be sold. This increases the availability of vegetables in Natitingou and may contribute to better consumption by children in this commune. Ethnicity has also been shown to exert an influence on students’ F&V consumption and the way they consume them, which could differ depending on their culture, ethnicity, or home practices. Similar results have been found by other authors [52,53].

### Limitations of the Study

Fruits and vegetables are seasonal, therefore influencing seasonal consumption and data collection. The F&V consumption appraised in this study is linked to the season in which the study was conducted and may not fully reflect all of the F&V eaten by the study cohort throughout the year. This is due to the project design. Furthermore, other sociocultural factors―such as parents’ education level, wealth, and culture/ethnicity―influencing F&V consumption have not been considered in the present study. Moreover, the 24-h recall did not collect quantitative data and therefore cannot provide an accurate overview of the real micronutrient intake of these students. Nonetheless, these limitations do not affect the reliability of the data presented in this paper.

The results of this study present a sex distribution that may not reflect the realities of other African countries, but in Benin, girls’ education is strongly promoted, which explains the high percentage of girl participants [54,55].

## 5. Conclusions

Fruit and vegetable consumption among school children has a direct bearing on micronutrient intake and related health benefits or risks. Our study aimed to quantify and assess F&V consumption among secondary school children—as part of the national/local school feeding programs—in two secondary schools in Boukombe and Natitingou, both food-insecure communities located in North Benin. Our findings confirm that F&V consumption in both study sites is below WHO’s recommendation of 400 g or five servings/portions of F&V per day. This should be considered a major risk factor for micronutrient deficiencies and related health risks and school performance among children/adolescents in these communities. These results should be an incentive for nutrition researchers, nutrition project managers, and national policies to adopt broader measures targeting the dietary practices of secondary school students in order to improve their F&V consumption. These should favor better availability of and accessibility to F&V in and around those schools, and culturally appropriate incentives to motivate adolescents to consume more F&V. One such initiative could be cultivating school gardens, which stimulate the children’s understanding and appreciation related to growing food, in addition to supporting positive/better nutrition outcomes. Interventions focusing or centered on women’s empowerment will also help mothers to improve their financial power that will in turn improve students’ access to healthy diets. Lastly, to appreciate the real micronutrient intake of these students and provide a more accurate overview of the nutritional situation, a quantitative 24-h recall should be conducted. Further surveys should be conducted at different times of the year to account for seasonal fluctuations in F&V availability.

## Figures and Tables

**Table 1:** Previous studies related to adolescents’ consumption of fruits and vegetables.

Type of Study	Location	Year	Target Population	Results	References
Cross-sectional study (review)	22 Arabic countries	2023	Adolescents 10–19 years	A low proportion (10–29%) of adolescents met the five recommended daily F&V servings/portions target, with lower consumption of fruits than vegetables.	[21]
Cross-sectional study	Bangladesh	2021	Secondary school students 14–18 years	Low intake was observed among secondary school students in Bangladesh, where only 21% ate at least five servings/portions of F&V per day.	[22]
Cross-sectional study	Ghana	2021	Secondary school students	The prevalence of adequate F&V intake (both eaten at least twice a day) among secondary school students was 35.7% and 26.8%, respectively, and adequate F&V intake (both eaten at least five times a day) was 27.8%.	[23]
Cross-sectional study	Benin	2019	Adolescents 10–19 years	Another study carried out in Cotonou on adolescents has shown that only 8.6% of adolescents/students consumed F&V at least five times a day.	[24]
Cross-sectional study	Benin	2010	Secondary school students 13–19 years	The average quantity of daily fruit and vegetable intake was 97 g.	[25]

**Table 2:** Overview of the study populations’ backgrounds.

Student Characteristics		Boukombe		Natitingou		*p*
		N	%	N	%	
Sex	Female	57	56.4	117	57.9	0.62
	Male	44	43.6	85	42.1	
Age (years, SD)		15.88 ± 2.72		15.32 ± 2.72		0.12
School grade	6th grade	28	27.8	37	18.3	<0.001
	7th grade	7	6.9	43	21.3	
	8th grade	18	17.8	21	10.4	
	10th grade	25	24.7	44	21.8	
	11th grade	23	22.8	57	28.2	
Father’s occupation	Farmer	45	44.7	35	17.3	<0.001
	Breeder	3	2.9	0	0	
	Public service worker	22	21.8	74	36.6	
	Taxi driver	4	3.9	13	6.4	
	Trader	4	3.9	19	9.4	
	Craftsman	8	7.9	40	19.9	
	Private service worker	13	12.9	9	4.4	
	Processor	2	2.0	6	3	
	No occupation	0	0	6	3	
Mother’s occupation	Farmer	13	12.9	9	4.4	<0.001
	Breeder	0	0	1	0.5	
	Public service worker	9	8.9	19	9.4	
	Trader	17	16.8	81	40.1	
	Craftsman	14	13.9	34	16.8	
	Caregiver	46	45.5	55	27.3	
	Private service worker	1	1	3	1.5	
	Processor	1	1	0	0	
Ethnic group	Ditamari	79	78.2	33	16.3	<0.001
	Bariba		-	29	14.4	
	Peulhs	2	2.0	2	1.0	
	Waama	2	2.0	27	13.4	
	Fon	3	2.9	18	8.9	
	Nago	1	1.0	4	2.0	
	Others	14	13.9	89	44.0	

Chi-square and Fisher test; *p*: *p*-value.; n = number; %: percentage.

**Table 3:** Fruit and vegetable consumption frequency.

Food Type	Consumption Frequency	Boukombe (n = 101)	Natitingou (n = 202)	*p*-Value
		%	%	
Fruits, excluding juice	2 or more times a day	8.9	11.0	0.01
	Once a day	12.9	20.0	
	5–6 times a week	2.9	2.0	
	3–4 times a week	13.9	17.5	
	1–3 times a month	2.9	11.0	
	Less than once a month or never	58.5	38.5	
Vegetables	2 or more times a day	9.9	11.4	0.12
	Once a day	12.9	21.4	
	5–6 times a week	1.9	3.5	
	3–4 times a week	20.9	21.9	
	1–3 times a month	2.9	7.0	
	Less than once a month or never	51.5	34.8	
Fruits, not including juice	2 or more times a day	8.9	11.0	0.01
	Once a day	12.9	20.0	
	5–6 times a week	2.9	2.0	
	3–4 times a week	13.9	17.5	
	1–3 times a month	2.9	11.0	
	Less than once a month or never	58.5	38.5	
Vegetables	2 or more times a day	9.9	11.4	0.12
	Once a day	12.9	21.4	
	5–6 times a week	1.9	3.5	
	3–4 times a week	20.9	21.9	
	1–3 times a month	2.9	7.0	
	Less than once a month or never	51.5	34.8	

Wilcoxon–Mann–Whitney test; n = number; %: percentage.

**Table 4:** Percentage of students based on the number of fruits and vegetables consumed per student.

			*p*-Value			*p*-Value	*p*-Value
	Boukombe		(SD#NSD)	Natitingou		(SD#NSD)	(Boukombe # Natitingou)
	SD (%)	NSD (%)		SD (%)	NSD (%)		
No. of different fruits consumed							0.037
0	84.84	79.79	0.385	83.63	71.55	0.067	
1	14.14	17.17		11.2	23.28		
2	1.0	3.0		4.56	4.63		
3	0	0		0.50	0.51		
No. of different vegetables consumed							<0.01
0	6.06	4.04	0.24	5.07	3.09	0.65	
1	15.15	12.12		8.12	8.76		
2	33.33	28.28		18.27	18.55		
3	20.20	26.26		17.76	19.58		
4	10.10	15.15		20.81	16.49		
5	9.09	8.08		13.19	13.91		
6	6.06	5.05		5.58	9.27		
7 or more	0	1.01		11.15	10.29		
Total	100	100		100	100		

(0) no fruit, (1) one fruit, (2) 2 fruits, (3) 3 fruits, (4) 4 fruits, SD = school day, NSD = non-school day. Wilcoxon–Mann–Whitney test. %: percentage.

**Table 5:** Factors associated with fruit consumption on school and non-school days.

	Estimate	Std. Error	Z Value	*p*
School day				
(Intercept)	−0.91691	1.15945	−0.791	0.43
Age	−0.45641	0.22998	−1.985	0.04
Sex	0.99633	0.36129	2.758	0.005
Commune Natitingou	0.44776	0.40890	1.096	0.27
Student ethnicity	−0.02352	0.01624	−1.449	0.14
Father’s occupation	−0.02601	0.08333	−0.312	0.75
Mother’s occupation	0.20452	0.09639	2.122	0.03
Non-school day				
(Intercept)	−32.8	2.140000	0.00	1.000
Age	−0.01	0.04	−0.30	0.76
Male	−0.33	0.26	−1.27	0.20
Commune Natitingou	−0.50	0.34	−1.45	0.14
Student ethnicity	2.68	0.86	3.12	0.001
Mother’s occupation	2.73	1.32	2.06	0.039

Poisson model with zero inflation; *p*: *p*-value.

**Table 6:** Factors associated with vegetable consumption.

	Estimate	Std. Error	Z Value	*p*
**(Intercept)**	3.703	0.714	26.85	<0.0001
**Age**	0.0110	0.0331	0.11	0.74
**Male**	−0.0325	0.1868	0.03	0.86
**Commune Boukombe**	−0.9814	0.2612	14.11	0.0001
**Student ethnicity**	−1.0168	0.4194	27.34	<0.0001

Poisson generalized estimating equations; *p*: *p*-value.

## Data Availability

The data and material used in this article are available and will be shared on request from the corresponding author, with the permission of the Alliance of Bioversity International and CIAT.
